# Supplementary material for: Tuning Electrostatic Gating of Semiconducting Carbon Nanotubes by Controlling Protein Orientation in Biosensing Devices
Source: Angew Chem Weinheim Bergstr Ger. 2021 Aug 6;133(37):20346–51. doi: 10.1002/ange.202104044 (PMC10946871; doi:10.1002/ange.202104044)
Supplement: Supplementary file 1 — Supporting Information [file ANGE-133-20346-s001.pdf]

Angewandte Chemie
Eine Zeitschrift der Gesellschaft Deutscher Chemiker

Supporting Information

Tuning Electrostatic Gating of Semiconducting Carbon Nanotubes by Controlling Protein Orientation in Biosensing Devices

Xinzhao Xu[+], Benjamin J. Bowen[+], Rebecca E. A. Gwyther, Mark Freeley, Bella Grigorenko, Alexander V. Nemukhin, Johnas Eklöf-Österberg, Kasper Moth-Poulsen, D. Dafydd Jones, and Matteo Palma**

ange_202104044_sm_miscellaneous_information.pdf

Supporting Information

1. Protein engineering and production.

Protein modelling to incorporate *p*-azido-L-phenylalanine (AzF) was performed as described previously [1-2]. Models of the protein-pyrene forms were built using optimised configurations of the AzF-DBCO-pyrene entity shown below. The AzF-DBCO-pyrene was constructed manually using the tools implemented in Accelrys Discovery Studio 2.5 (Accelrys Inc.). Optimization of geometry parameters of selected conformations was performed by quantum chemical calculations with the Firefly program package (http://classic.chem.msu.su/gran/firefly/index.html [3]) at the density functional theory level using the PBE0 functional [4] and cc-pVDZ basis set. The molecule is a highly flexible system with numerous conformations corresponding to different local minima on the potential energy surface.

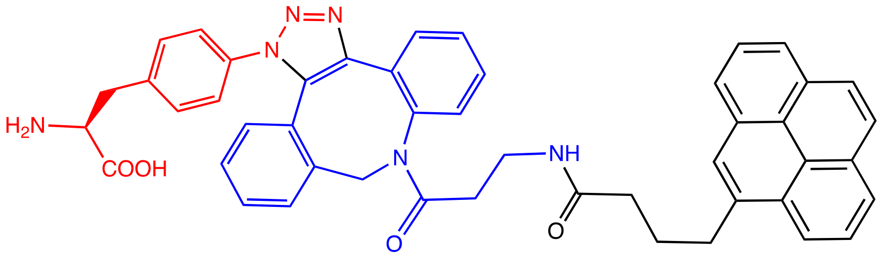

In this particular application, in which this molecule serves as a linker between the nanotube and the protein, some conformations are not suitable candidates as steric clashes with BLIP2 itself and between BLIP2 and tube will rule them out. Therefore, reasonable constructs were manually selected among the entire set, with the allowable lowest energy form outlined in Figure 4b in the main text. The AzF-DBCO-pyrene molecule was then incorporated into BLIP2 using the original AzF models for each variant with the FUSE command within PyMOL (Schrödinger) used to attach the rest of the DBCO-pyrene. The pyrene moiety was then manually docked to SWCNTs based on the well-known pi-pi stacking interaction. Electrostatic surfaces were calculated using the Adaptive Poisson-Boltzmann Solver (ABPS)[5] Electrostatics Plugin for PyMol.

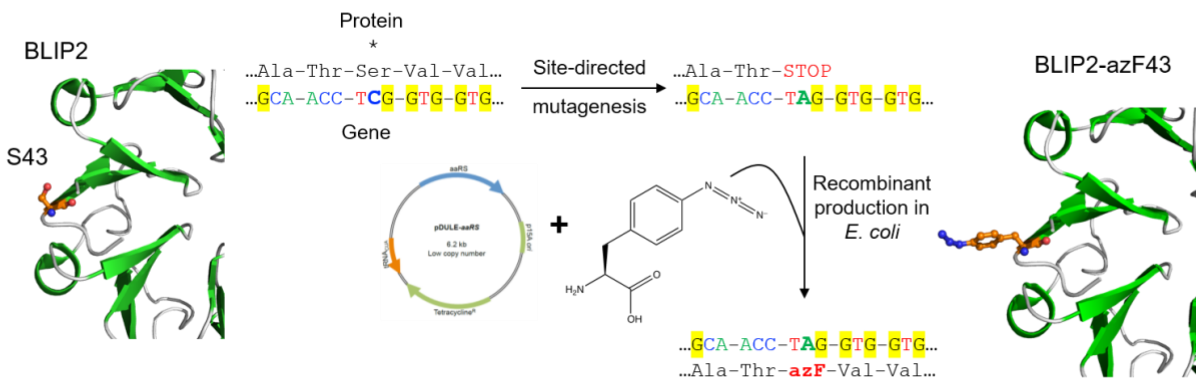

Scheme SI-1. Engineering of proteins with the noncanonical amino acid (ncAA) azF (p-azido-L-phenylalanine) at defined sites/residues via a reprogrammed genetic code approach

Site directed mutagenesis and incorporation was performed as outlined in Scheme S1 above. Details for BLIP^{41AzF} have been presented elsewhere [2] and are outlined here as part of the general approach for generating the BLIP-AzF mutants.

The BLIP2-azF mutants were constructed using the primers sets given below. PCR was performed with NEB Q5 polymerase according to manufacturer's directions. PCR products were then purified using the QIAgen PCR purification kit. Purified PCR products were recircularised with T4 PNK and Quick ligase (both NEB). DNA sequences were verified by sequencing provided by Eurofins Genomics and are presented below.

	**Sequence (5' - 3')**
**A41TAG mutation**	TATACATATGtagACCTCGGTGGTG<br>TCTCCTTCTTAAAGTTAAACAAAATTATTTCTA
**G49TAG mutation**	CCTGGGGCtagAACAATGACTG<br>CCACCACCGAGGTTGCCATA
**T213TAG mutation**	TTGGCCAGACCtagGTGC<br>AATAATTATCCCCCCACGCAATAACAC

S43TAG gene sequence

ATGGCAACC**TAG**GTGGTGGCCTGGGGCGGTAACAATGACTGGGGTGAAGCTACCGTGCCGGCC
GAAGCGCAGAGTGGTGTGGATGCAATTGCAGGTGGTTATTTTCATGGGCTGGCACTGAAAGGGG
GTAAAGTACTGGGCTGGGGTGCAAATCTGAACGGGCAGCTGACAATGCCGGCGGCGACCCAGA
GCGGCGTTGATGCTATCGCGGCGGGCAATTATCACTCTCTGGCTCTGAAAGATGGGGAAGTGAT
TGCTTGGGGCGGTAACGAGGATGGCCAAACTACGGTGCCGGCCGAGGCCCGTTCCGGTGTAGA
TGCTATTGCGGCAGGCGCTTGGGCGAGCTACGCGCTGAAAGACGGCAAAGTGATCGCCTGGGG
TGATGATTCCGACGGTCAGACCACCGTGCCGGCGGAAGCCCAGTCGGGTGTGACCGCGCTGGA
TGGTGGTGTGTATACCGCGCTGGCAGTAAAAAACGGTGGTGTTATTGCGTGGGGGGATAATTAT
TTTGGCCAGACCACAGTGCCGGCGGAGGCTCAGTCCGGGGTGGATGATGTTGCAGGCGGCATC
TTTCACAGCCTGGCGCTGAAAGATGGTAAAGTTATTGCGTGGGGCGATAATCGCTATAAACAAAC
CACAGTTCCAACCGAGGCGCTGAGTGGCGTGTCGGCCATTGCTTCAGGTGAATGGTATAGCCTG
GCTCTGAAAAATGGTAAAGTAATTGCGTGGGGTAGCAGCCGCACCGCGCCTAGCTCCGTCCAAT
CGGGGGTGAGTTCCATTGAAGCCGGTCCGAACGCCGCTTACGCACTGAAAGGTGGGAGCGGTT
CTGGCCATCATCACCATCATCATTAA

G49TAG gene sequence
ATGGCAACCTCGGTGGTGGCCTGGGGC**TAG**AACAATGACTGGGGTGAAGCTACCGTGCCGGCC
GAAGCGCAGAGTGGTGTGGATGCAATTGCAGGTGGTTATTTTCATGGCTGGCACTGAAAGGGG
GTAAAGTACTGGGCTGGGGTGCAAATCTGAACGGGCAGCTGACAATGCCGGCGGCGACCCAGA
GCGGCGTTGATGCTATCGCGGCGGGCAATTATCACTCTCTGGCTCTGAAAGATGGGGAAGTGAT
TGCTTGGGGCGGTAACGAGGATGGCCAAACTACGGTGCCGGCCGAGGCCCGTTCCGGTGTAGA
TGCTATTGCGGCAGGCGCTTGGGCGAGCTACGCGCTGAAAGACGGCAAAGTGATCGCCTGGGG
TGATGATTCCGACGGTCAGACCACCGTGCCGGCGGAAGCCCAGTCGGGTGTGACCGCGCTGGA
TGGTGGTGTGTATACCGCGCTGGCAGTAAAAAACGGTGGTGTTATTGCGTGGGGGGATAATTAT
TTTGGCCAGACCACAGTGCCGGCGGAGGCTCAGTCCGGGGTGGATGATGTTGCAGGCGGCATC
TTTCACAGCCTGGCGCTGAAAGATGGTAAAGTTATTGCGTGGGGCGATAATCGCTATAAACAAAC
CACAGTTCCAACCGAGGCGCTGAGTGGCGTGTCGGCCATTGCTTCAGGTGAATGGTATAGCCTG
GCTCTGAAAAATGGTAAAGTAATTGCGTGGGGTAGCAGCCGCACCGCGCCTAGCTCCGTCCAAT
CGGGGGTGAGTTCCATTGAAGCCGGTCCGAACGCCGCTTACGCACTGAAAGGTGGGAGCGGTT
CTGGCCATCATCACCATCATCATTAA

S213TAG gene sequence
ATGGCAACCTCGGTGGTGGCCTGGGGCGGTAACAATGACTGGGGTGAAGCTACCGTGCCGGCC
GAAGCGCAGAGTGGTGTGGATGCAATTGCAGGTGGTTATTTTCATGGCTGGCACTGAAAGGGG
GTAAAGTACTGGGCTGGGGTGCAAATCTGAACGGGCAGCTGACAATGCCGGCGGCGACCCAGA
GCGGCGTTGATGCTATCGCGGCGGGCAATTATCACTCTCTGGCTCTGAAAGATGGGGAAGTGAT
TGCTTGGGGCGGTAACGAGGATGGCCAAACTACGGTGCCGGCCGAGGCCCGTTCCGGTGTAGA
TGCTATTGCGGCAGGCGCTTGGGCGAGCTACGCGCTGAAAGACGGCAAAGTGATCGCCTGGGG
TGATGATTCCGACGGTCAGACCACCGTGCCGGCGGAAGCCCAGTCGGGTGTGACCGCGCTGGA
TGGTGGTGTGTATACCGCGCTGGCAGTAAAAAACGGTGGTGTTATTGCGTGGGGGGATAATTAT
TTTGGCCAGACC**TAG**GTGCCGGCGGAGGCTCAGTCCGGGGTGGATGATGTTGCAGGCGGCATC
TTTCACAGCCTGGCGCTGAAAGATGGTAAAGTTATTGCGTGGGGCGATAATCGCTATAAACAAAC
CACAGTTCCAACCGAGGCGCTGAGTGGCGTGTCGGCCATTGCTTCAGGTGAATGGTATAGCCTG
GCTCTGAAAAATGGTAAAGTAATTGCGTGGGGTAGCAGCCGCACCGCGCCTAGCTCCGTCCAAT
CGGGGGTGAGTTCCATTGAAGCCGGTCCGAACGCCGCTTACGCACTGAAAGGTGGGAGCGGTT
CTGGCCATCATCACCATCATCATTAA

To recombinantly produce the BLIP2-AzF variants, *E. coli* BL21 (DE3) cells (NEB) were transformed with the pET-BLIP2 (kindly provided by Tim Palzkill) and pEVOL-AzF plasmids (provided by Ryan Mehl via Addgene) and grown on LB agar supplemented with 30 µg/mL kanamycin and 35 µg/mL choramphenicol for transformant selection; colonies were picked and grown in 500 mL 2×YT broth with 30 µg/mL kanamycin and 35 µg/mL choramphenicol at 37 °C until reaching an OD600 of 0.4. AzF and IPTG were added, both to 1 mM to induce expression and the cells grown for a further 24 hours at 16 °C with 200rpm shaking. BLIP2^{WT} was produced in a similar manner but without antibiotic and AzF additions required to cultivate cells transformed with pEVOL-AzF [2]. The cells were pelleted by centrifugation and the cells resuspended in binding buffer (50 mM Tris-HCl, 5 mM imidazole pH8.0). The cells were lysed using a French Press exerting 20,000 psi of pressure. Soluble protein was harvested by centrifugation at 25,000 x g for 10 mins. The supernatent containing histidine-tagged BLIP2 were first purified by cobalt affinity chromatography using a home-made gravity column containing 5mL HisPur cobalt resin (Thermofisher) equilibrated in binding buffer (50 mM Tris-HCl, 5 mM imidazole pH8.0). The column was then washed with 10 column volumes of binding buffer before being eluted in a single step with 20mL 100 mM imidazole, then concentrated and desalted using a PD10 column (GE). Purity of protein was assessed by SDS-PAGE.

2. Enzyme inhibition by BLIP2 and its AzF variant.

The BL assay measured the initial rate of nitrocefin hydrolysis by TEM^{WT}, by recording the absorbance increase on hydrolysis of the β-lactam ring amide bond. Initial rates were recorded using 0.3 nM TEM with increasing concentrations of BLIP2 until full inhibition had been achieved for all BLIP2 variants. The initial rates were plotted and fitted to the Morrison Equation [6-7] using GraphPad Prism software to estimate the K_i^{app} for each interaction.

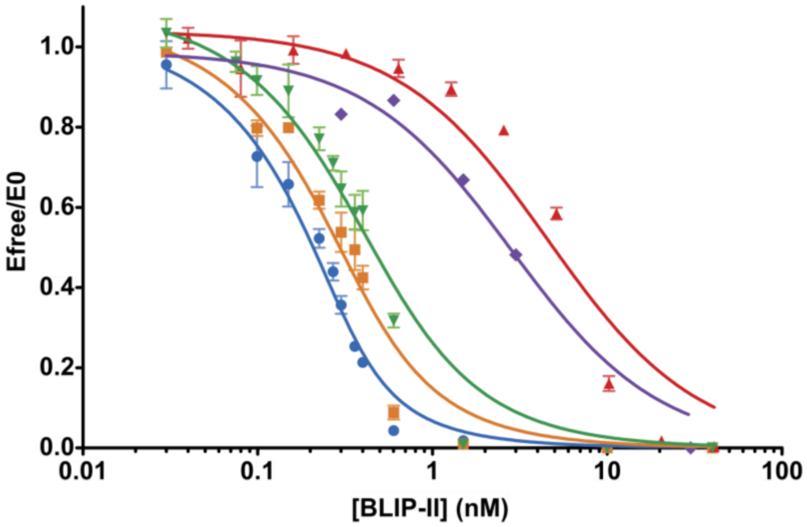

Figure SI-1. Inhibition of TEM^{WT} by BLIP2 variants. In order of binding affinity, left to right: Blue: $BLIP2^{WT}$. Orange: $BLIP2^{41AzF}$. Green: $BLIP2^{213AzF}$. Purple: $BLIP2^{43AzF}$. Red: $BLIP2^{49AzF}$. Values were fitted to the Morrison tight binding equation to calculate K_i^{app}. E_{free}/E_0 is the proportion of free enzyme remaining. Calculated K_i^{app} for each variant: $BLIP2^{WT}$ 32 ± 4 pM; $BLIP2^{41AzF}$ 78 ± 12 pM; $BLIP2^{43AzF}$ 1724 ± 378 pM; $BLIP2^{49AzF}$ 2839 ± 406 pM; $BLIP2^{213AzF}$ 157 ± 20 pM.

Additional explanatory methods and text.

To quantitatively determine the effect of the four mutations on BLIP2's ability to bind (and thus inhibit) TEM^{WT}, enzyme inhibition assays were used to measure the apparent inhibition constants (K_i^{app}) compared to $BLIP2^{WT}$. K_i is equivalent to dissociation constant (K_D) but in the context of enzyme inhibition. Absolute binding parameters for BLIP2-BL interactions are difficult to determine by normal methods as their dissociation constant is so low (80-540 fM). Working at protein concentrations in this range are impractical for our requirements so a comparative analysis with $BLIP2^{WT}$ will allow an apparent K_i to be determined. This will allow the direct effect of the mutation on binding affinity to be assessed. The $BLIP2^{WT}$-TEM^{WT} dissociation constant (K_D) has previously been determined to be 480 fM using separately measured association and dissociation rate constants. However, these experiments require up long term (weeks) incubation of $BLIP2^{WT}$-TEM^{WT} complexes in an excess of the inactive TEM^{166Ala} mutant and was considered unnecessary for our purpose of comparing the variants to $BLIP2^{WT}$.

This allowed us to investigate the binding affinities of the BLIP2 variants for the wild-type BL enzyme TEM used in the electrical measurements. $BLIP2^{WT}$ (wild type) has a

high affinity for TEM-1 BL (BL^{TEM}), which was also observed here (K_i 32 ± 4 pM). Both $BLIP2^{41AzF}$ and $BLIP2^{213AzF}$ retain sub-nanomolar affinity for TEM (K_i 78-160 pM). As predicted, $BLIP2^{49AzF}$ exhibited reduced binding affinity (K_i 2839 ± 406 pM) due to its location at the BLIP2-BL binding interface.

3. Electrode fabrication and protein attachment

Electrodes were prepared as previously reported.[8] Nanosized electrodes were fabricated on a p-doped Si/SIO_2 wafer by a combination of laser and electron beam lithography, followed by evaporating a thin adhesive layer of Cr and a thick layer of Au.

95% semiconducting single walled carbon nanotubes were purchased from Nanointegris. DBCO-amine and pyrene-NHS were purchased from Sigma-Aldrich. All of other chemicals used in this paper were ordered from Sigma-Aldrich.
0.1 mg 95% semiconducting SWNT was dispersed in 500 µL of 1% SDS solution via sonication for 1h. The supernatant of SWNT was collected after being centrifuged for 1h, which was used as stock solution.
To immobilise a small bundle of SWNTs between electrodes, dielectrophoresis (DEP) was performed by applying an alternating current (AC) voltage between electrodes after SDS-dispersed SWNT solution was cast on the electrodes. Typically, the frequency of the generator was switched onto V_{p-p} = 3V at f=400KHz.
The as prepared CNT solution was diluted by 100-fold and cast onto the chip with a pipette (5 µL). After a delay of 10 seconds, the substrate was washed carefully with water to remove SDS and blown gently with Nitrogen gas. Electrical measurement was performed to confirm the immobilisation of SWNTs between electrodes.
0.4mg 1-Pyrenebutyric acid N-hydroxysuccinimide ester (pyrene-NHS) and 0.5mg Dibenzocyclooctyne-amine (DBCO-amine) were dissolved and mixed in 200 µL dimethylformamide (DMF) to give a solution containing 5 mM of pyrene-NHS and 9mM of DBCO-amine. The mixture was placed on a shaker overnight at room temperature to form DBCO-pyrene. Subsequently, 10 µL ethanol-amine was added to the mixture to blocked unreacted NHS groups.
The prepared devices were immersed in the mixture solution for 1h. DBCO-pyrene would be immobilised onto the sidewall of SWNT bundles via π-π stacking between pyrene and SWNTs. The devices were rinsed with iso-propanol and DPBS buffer after incubation. Subsequently, 20 µL azide modified BLIP2 variants (200 nM, in DPBS) were cast on the devices. After incubation for 3h, the devices were rinsed with water and blown dried with Nitrogen gas for atomic force microscopy (AFM) imaging. For electrical measurements, pyrene-butanol (PB, ratio of PB to pyrene-NHS is 3:1) was added into the mixture solution after the formation of DBCO-pyrene, serving as spacers to create enough space for the binding of BLIP2 to TEM. Additionally, after incubation for 3h, the devices were rinsed with DPBS buffer and used to performed real time detection of TEM. The biosensing electrical measurements were performed the same day of protein interfacing to minimise the potential degradation/instability of the protein-CNT hybrids over time

4.Characterisation

4.1 AFM characterisation

Topography analysis of the electrodes were imaged with a Bruker Dimension Icon atomic force microscope (AFM) with ScanAsyst Air tips. The images were analysed with NanoScope Analysis.

4.2 Electrical measurement

Electrical measurements were performed using a probe station (PS-100, Lakeshore) equipped with a semiconducting parameter analyser (Keithley, 4200SCS) at room temperature. I-V curves were measured with bias sweeping mode (-0.5 V to 0.5V) applied to source electrode while drain electrode was grounded. Transfer characteristics were measured with gate bias sweeping mode (-15 V to 15 V) to record the source-drain current (I_{sd}) versus gate bias (V_g) data.

4.3 Real time detection

For real time measurements, 100 mV source-drain bias were applied across the devices (gate electrode was grounded) which have been covered with a drop of DPBS buffer solution. DPBS buffer was first added to the devices after the reading of the current was stable, which cannot induce changes in current. This stable reading of the current through devices was recorded as background. Subsequently, 5µL of TEM solutions of various concentrations, were cast on the devices.

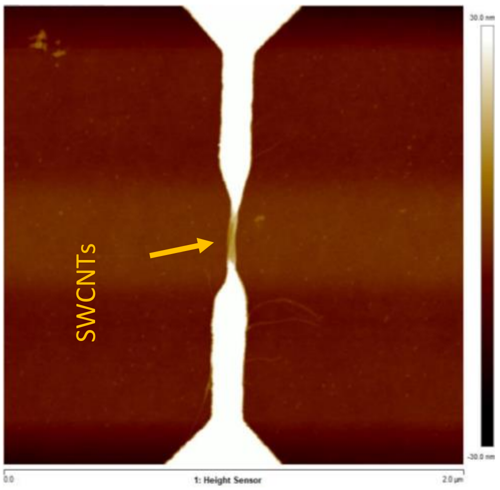

Figure SI-2. Representative AFM image of device after DEP, showing a small bundle of CNTs have been immobilised between electrodes

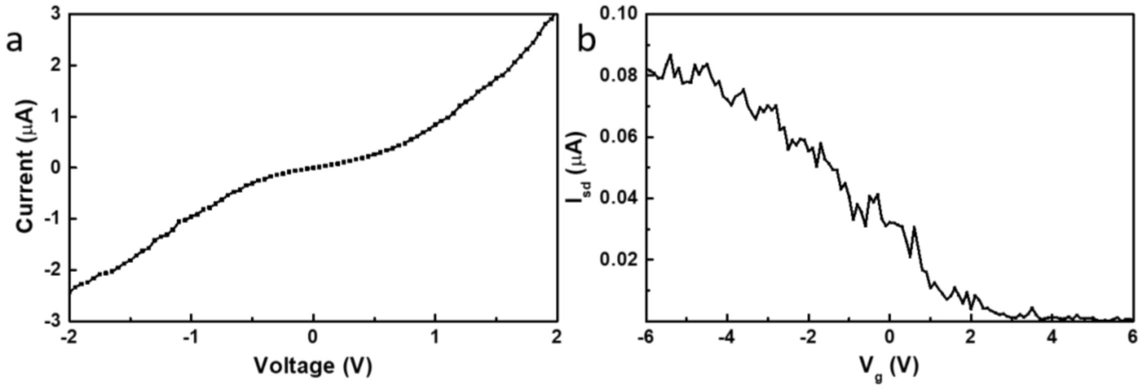

Figure SI-3. Representative (a) I-V curve and (b) transfer characteristics of the device (V_{sd}=0.1V)

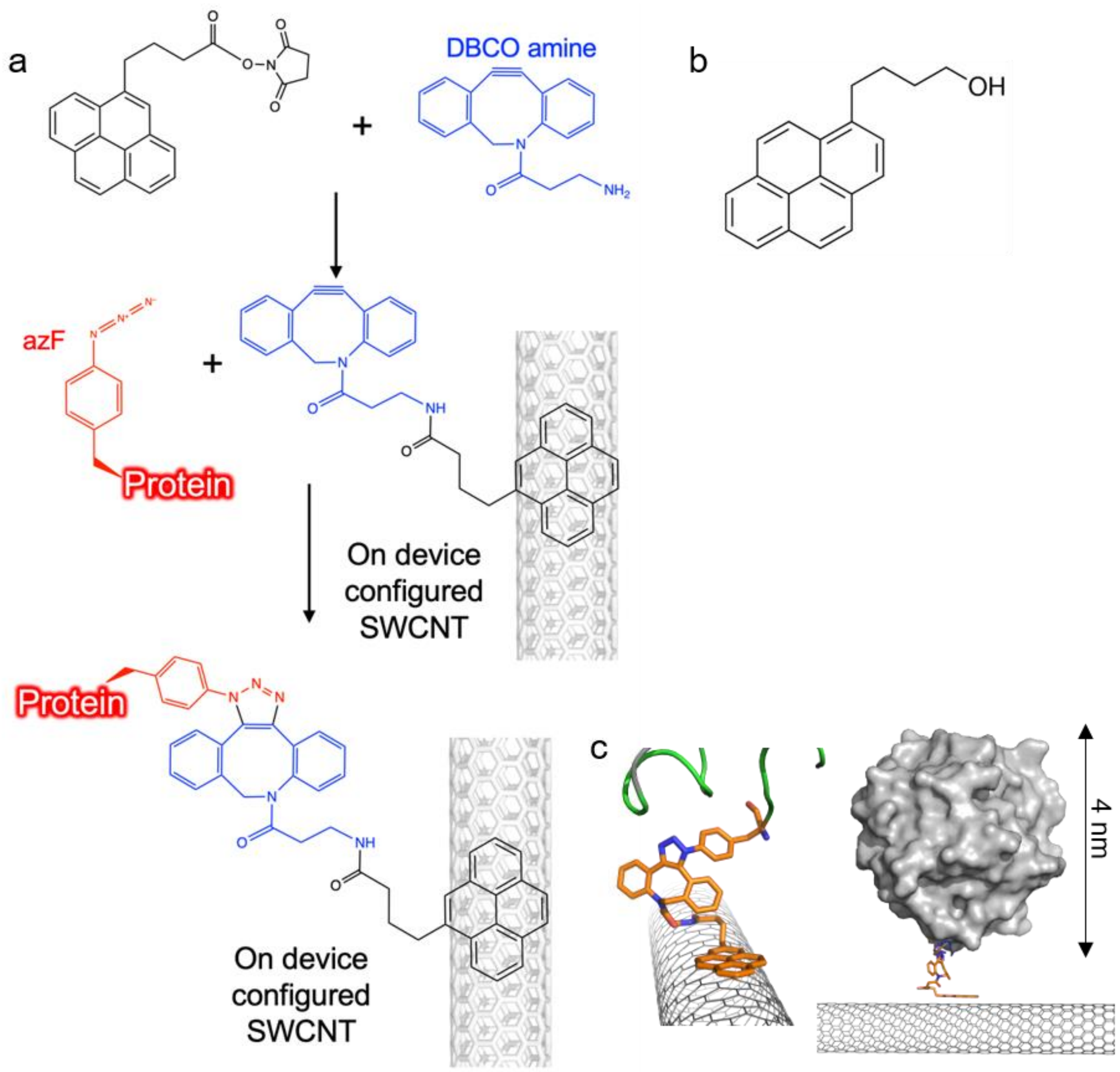

Figure SI-4. (a) Outline of approach for attachment of proteins to SWCNTs (b) Pyrene-butanol has a similar molecular structure to pyrene-NHS, which can bind to SWCNTs as well. Hence, we used it as spacers. Additionally, we used it to rule out non-specific adsorption of proteins to SWCNTs (c) Model of the BLIP2^{41AzF}-SWCNT interface, with a close-up of the pyrene-protein conjugation site.

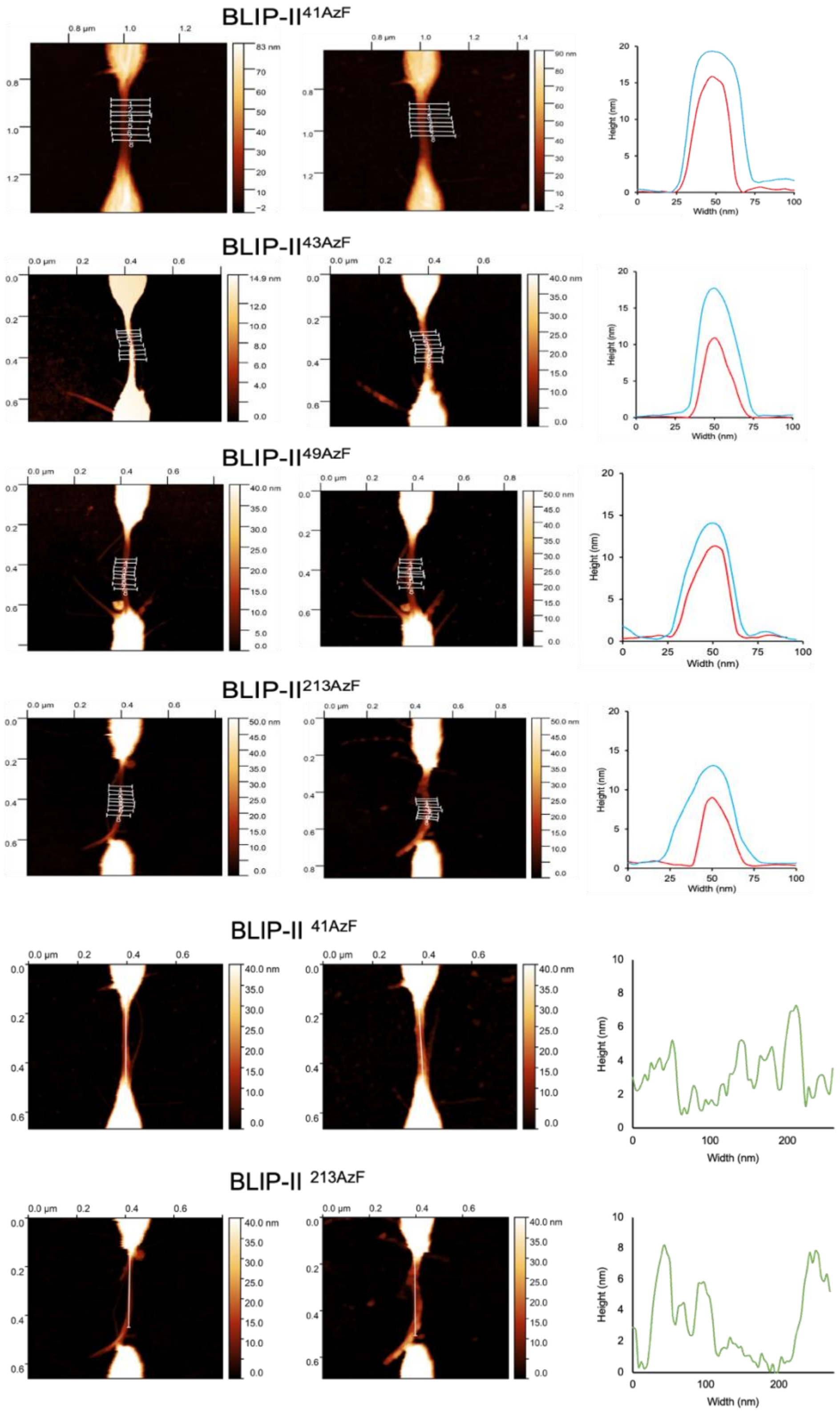

Figure SI-5. Functionalisation of CNT devices with BLIP2 AzF proteins. AFM topographical images and transverse height profiles across DBCO-pyrene functionalised CNT bundles before (left AFM images, red line) and after (right AFM images, blue line) protein functionalisation; profiles taken along the CNTs are shown as normalised height profiles in green for CNT bundles with and without BLIP2^{41AzF} and BLIP2^{213AzF}: in the normalised profiles the height of the original CNTs has been

subtracted from the CNTs + protein profile. Average bundle heights increased for all variants after incubation with proteins.

We counted the proteins on individual CNTs/small bundles from AFM topographical images (Figure SI-6). We estimated that there are 2.6 ± 0.7 proteins attached to an individual CNT per 100 nm. This is similar coverage to that observed on using the photo-induced UV-nitrene aziridine functionalisation.[2] To estimate the number of proteins per device, we also calculated the surface area of CNT bundles in each device compared to individual CNTs. Since the height of CNT bundles between electrodes is about 10-20nm, we estimate that there are 10-20 CNTs immobilised in each device. Bundles of CNTs containing 10 or 20 CNTs were modelled on a flat surface using PyMOL and their solvent-accessible surface area measured using the “get_area” command. It was calculated that a 10-CNTs bundle has ~ 5.5 times the available area of an individual CNT on a flat surface and a 20-CNT bundle has ~ 9.3 times the area. The gap between gold electrodes in the FET devices is 300nm. Therefore, we estimate that the number of proteins attached in each device is of 40 to 80 proteins.

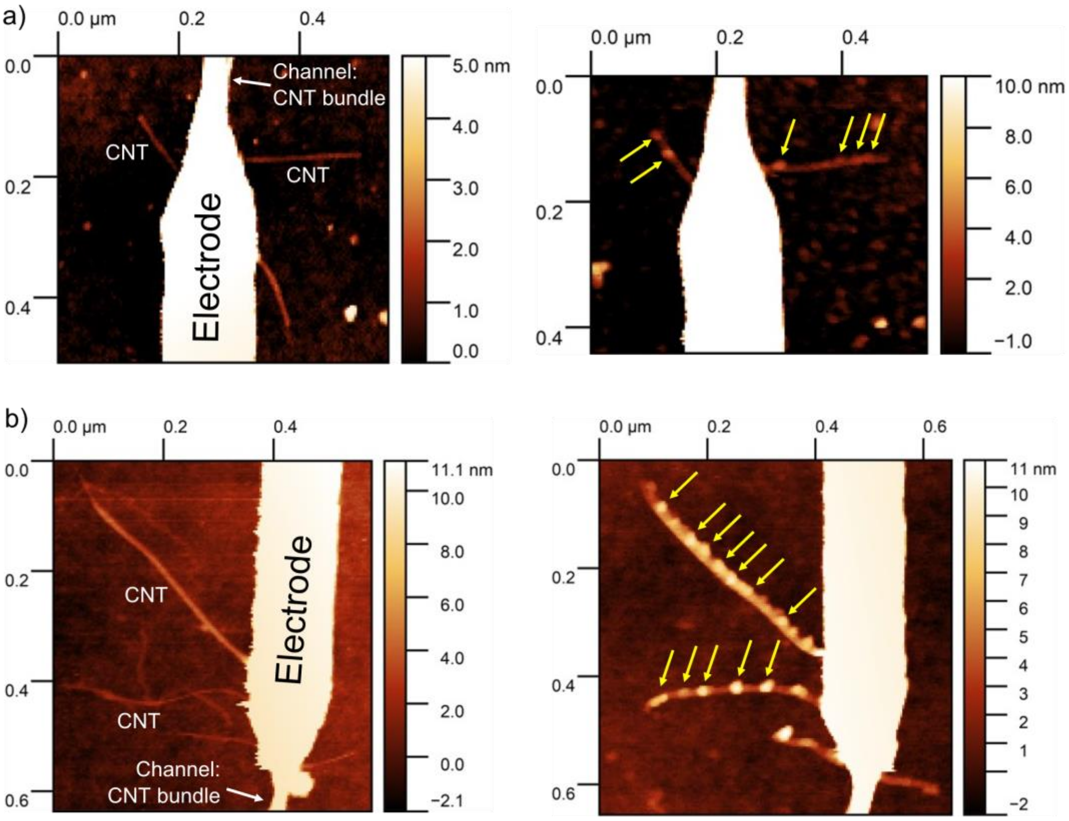

Figure SI-6. Representative AFM topographical images showing the attachment of proteins on individual CNTs/small bundles. The arrows indicate the location of proteins attached.

5. Control experiments with BLIP^{WT} and pyrene-butanol

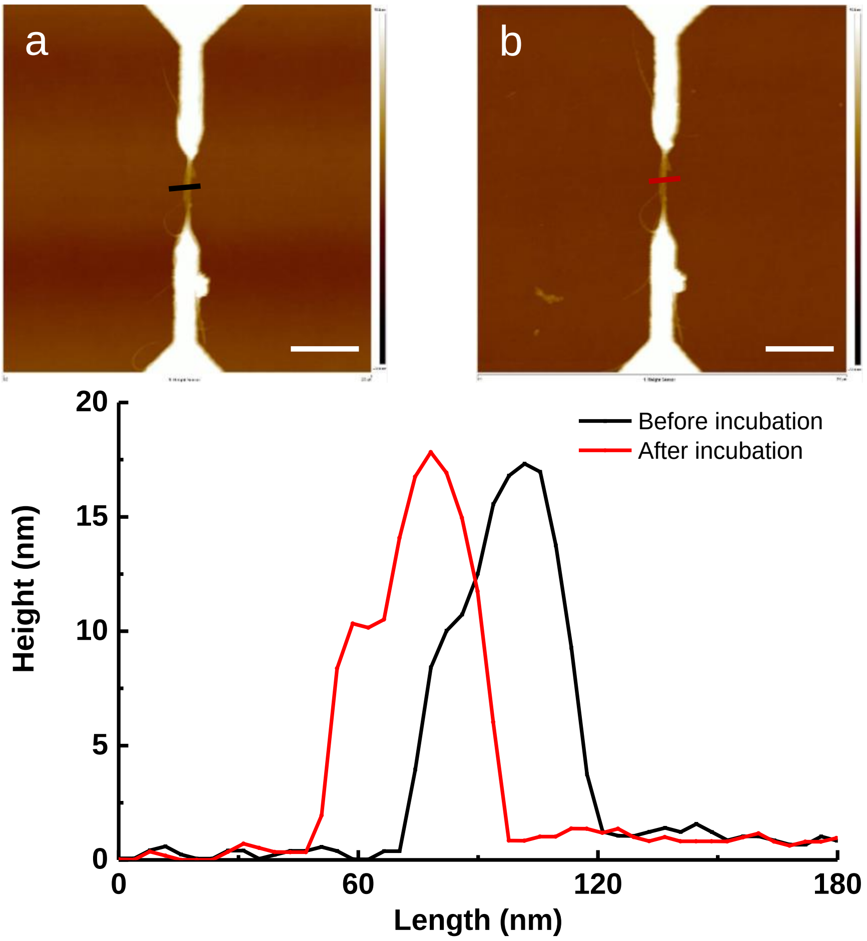

Figure SI-7. Control experiment with BLIP^{WT}. AFM topographical images of devices (a) before incubation with BLIP^{WT} (17-18nm) and (b) after incubation with BLIP^{WT} (17-18nm) and their corresponding height profiles. (scale bar: 400nm). This demonstrates that devices functionalised with DBCO groups can only react with AzF modified BLIP2.

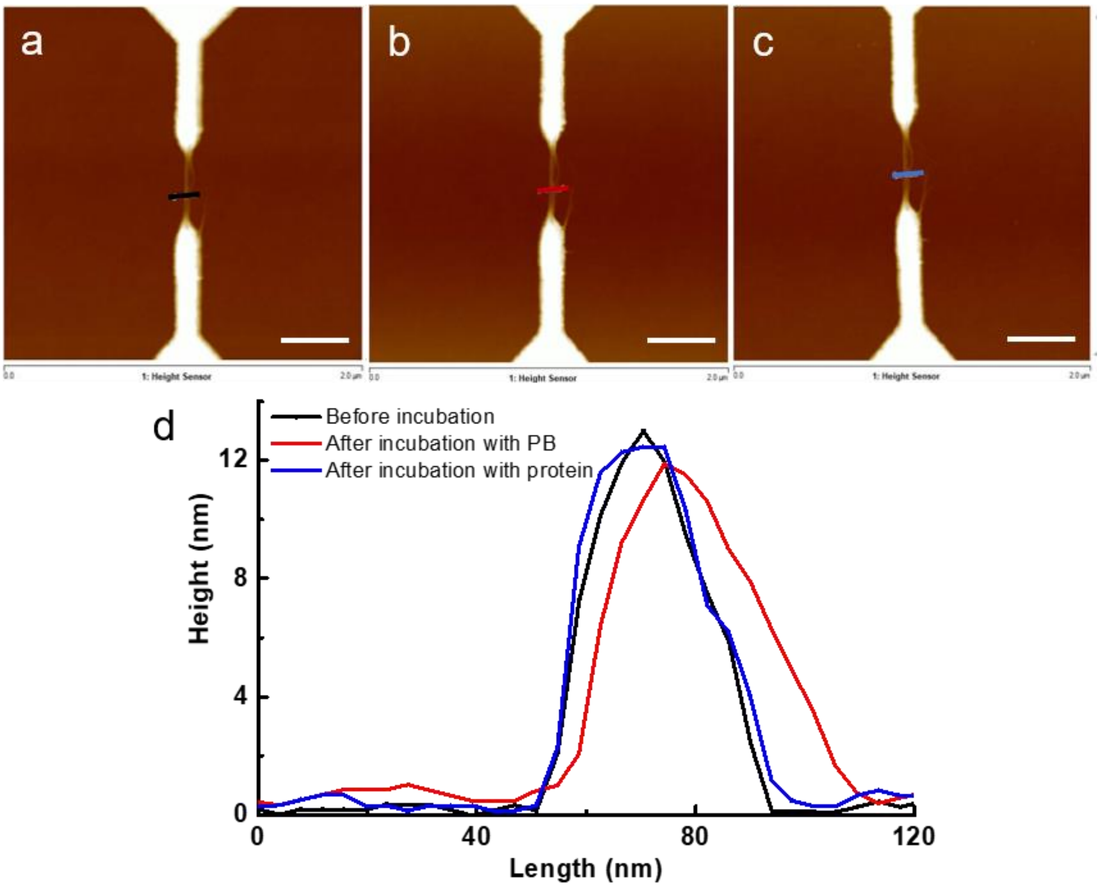

Figure SI-8. AFM topographical images of devices (a) before incubation with PB (12-13nm) (b) after incubation with PB (12-13nm) (c) after incubation with BLIP2^{213AzF} (12-13nm). (scale bar:400 nm). (d) topographcial profiles from the AFM iamges. This indicates that pyrene groups do not induce changes in height and hence that DBCO groups are essential for the attachment of BLIP2.

6. Calculation of Debye Length of 1×DPBS solution

According to the protocol from the company, the main components of 1×DPBS solution is sodium phosphate (Na_2HPO_4), sodium chloride (NaCl). 1×DPBS contains c.a. 137mM NaCl and 8 mM Na_2HPO_4. According to Formula (1), the ionic strength (I) is:

Ionic Strength: $$I = \frac{1}{2}\sum c_i \, {z_i}^2 \qquad (1)$$

where c_i is the molar concentration of ion i (M, mol/L), z_i is the charge number of that ion, and the sum is taken over all ions in the solution. Therefore, the ionic strength of 1×DPBS is:

$$I = 0.5 \times (0.137 \times 1^2 + 0.137 \times 1^2 + 0.03 \times 1^2 + 0.01 \times 3^2)\ \text{M}$$

$$I = 0.20\ \text{M}$$

Based on Formula (2), the Debye Length (λ_D) is:

Debye Length: $$\lambda_D \approx {0.32}/{\sqrt{I}} \qquad (2)$$

Therefore, the Debye Length of 1×DPBS IS:

$$\lambda_D \approx 0.7\ \text{nm}$$

7. Real time detection of TEM in serum

We also performed real time detection of TEM in serum. Steroid-free serum purchased from MP Biomedicals, Inc. was diluted with the DPBS buffer by 10-fold (serum solution). TEM solutions used in this detection was diluted with serum solution. 100 mV bias were applied between source-drain electrodes (gate electrode was grounded). A drop of serum solution (10µL) was drop-cast on the devices: this volume was chosen also due to the miniaturized size of the device (please note that evaporation of liquid can affect the electrical measurements if these are performed in a prolonged time) . Serum solution was added to the devices when the reading of the current was stable. Subsequently, 5µL of TEM solutions (diluted with serum solution) of various concentrations, were cast on the devices.
Although transfer characteristics (Isd vs Vg) can provide information about the electronic behavior of the CNTs, we preferred to present the real time biosensing measurements as more relevant - and in our case more accurate - to demonstrate the sensing results of the platform we developed. This because we found that many factors can influence the accuracy of Isd vs Vg data, from the evaporation of liquid affecting the concentration of solution, to issues in the devices due to the deposition of CNT bundles.

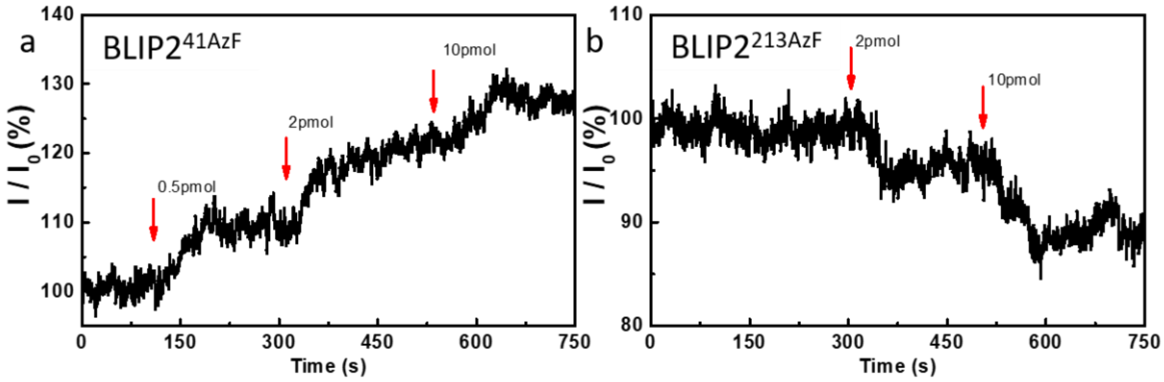

Figure SI-9. Real time detection of TEM-1 in serum using devices with (a) BLIP2^{41AzF} and (b) BLIP2^{213AzF} that we found to be marginally less sensitive in serum

8. Control experiments with non-functionalised devices

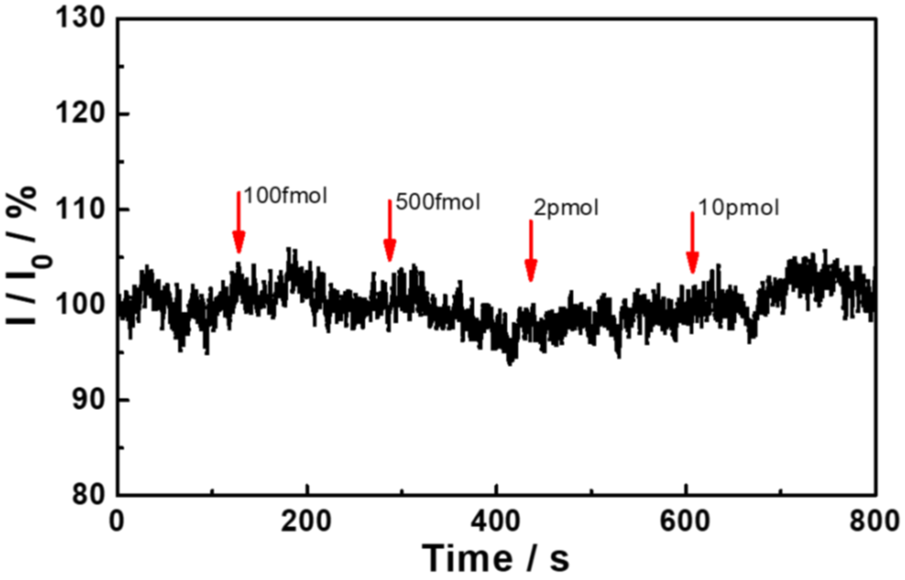

Figure SI-10. Real time detection of TEM-1 with non-functionalised devices

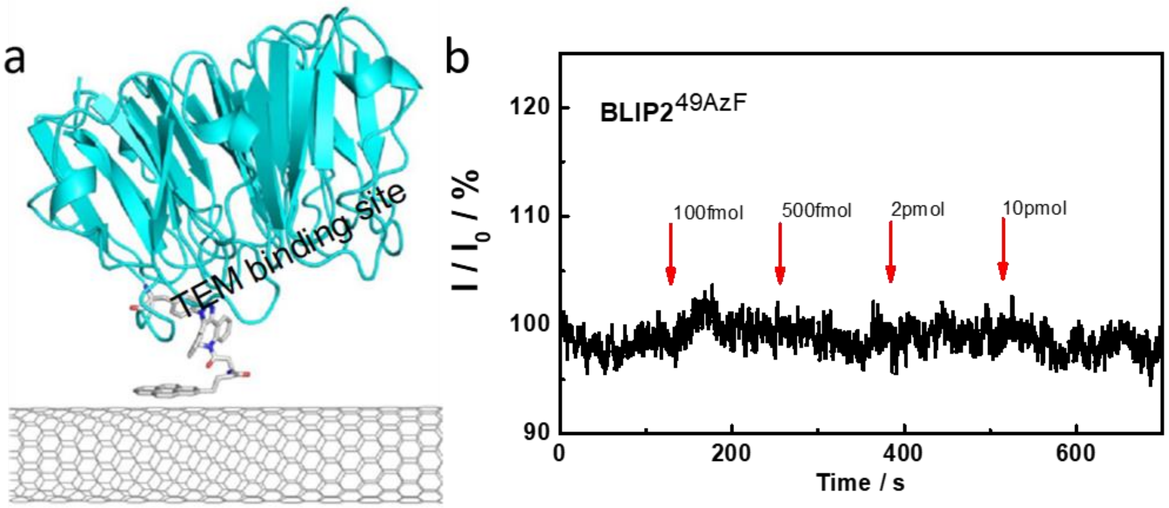

Figure SI-11. (a) Model of the BLIP2^{49AzF}-SWCNT complex and (b) Conductance across SWCNTs electrodes functionalised with BLIP2^{49Az}F. The red arrows indicate the time points when TEM at the stated concentration was added. The binding site for incoming TEM will be occluded by both the SWCNT and the pyrene adduct.

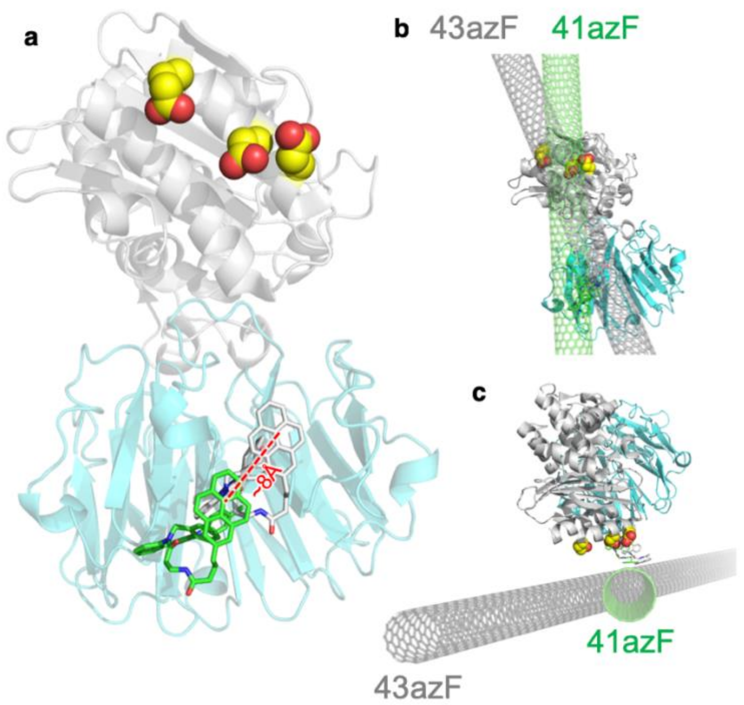

Figure SI-12. Modelling the potential orientation difference of BLIP2^{43AzF} *versus* BLIP2^{41AzF} on CNTs. (a) structural alignment of TEM-BLIP complex that highlights the change in position of the pyrene moiety (grey for BLIP^{43AzF}, green for BLIP^{41AzF}). There is ~8 Å shift in the position of the pyrene moiety. The acidic residue comprising the acidic patch highlighted in the manuscript are coloured yellow. (b) The effect of changing AzF location on the relative placement of TEM with respect the CNT. The relative change in the CNT position is shown for contrast. There is a ~21° change in the relative position of the CNTs and the key acidic residues appear to be further away (by 1-2 Angstroms) so taking them just beyond the calculated Debye field. (c) side-on view of that shown in (b). As well as the orientation aspects outlined here, the lower affinity of BLIP^{43AzF} for TEM (see Figure SI-1) may also contribute to the reduced signal change observed.

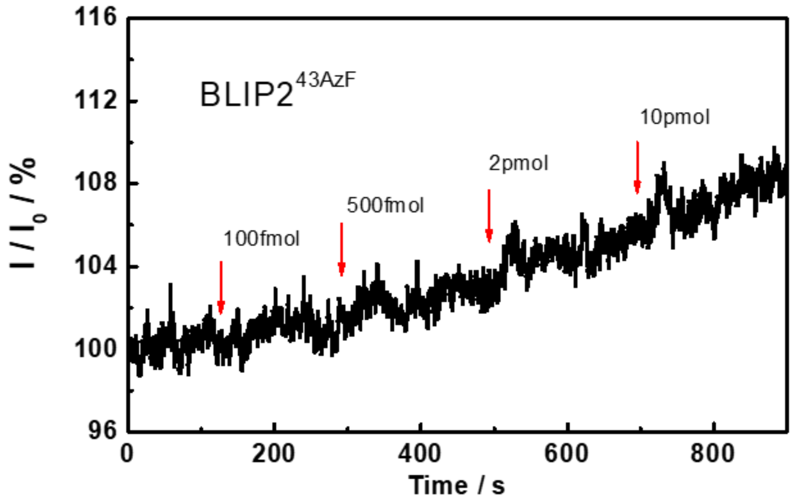

Figure SI-13. Real time detection of TEM-1 using devices with BLIP2^{43AzF}

References

(1) Hartley, A. M.; Zaki, A. J.; McGarrity, A. R.; Robert-Ansart, C.; Moskalenko, A. V.; Jones, G. F.; Craciun, M. F.; Russo, S.; Elliott, M.; Macdonald, J. E.; Jones, D. D., Functional Modulation and Directed Assembly of an Enzyme Through Designed Non-natural Post-translation Modification. *Chem Sci* **2015**, *6* (7), 3712-3717.

(2) Thomas, S. K.; Jamieson, W. D.; Gwyther, R. E. A.; Bowen, B. J.; Beachey, A.; Worthy, H. L.; Macdonald, J. E.; Elliott, M.; Castell, O. K.; Jones, D. D., Site-Specific Protein Photochemical Covalent Attachment to Carbon Nanotube Side Walls and Its Electronic Impact on Single Molecule Function. *Bioconjug Chem* **2020**, *31* (3), 584-594.

(3) Granovsky, A. A. Firefly version 8. http://classic.chem.msu.su/gran/firefly/index.html.

(4) Adamo, C., Oward Reliable Density Functional Methods without Adjustable Parameters: The PBE0 model. *J Chem Phys* **1999**, *110*, 6158-6170.

(5) Jurrus, E., Engel, D., Star, K., Monson, K., Brandi, J., Felberg, L.E., Brookes, D.H., Wilson, L., Chen, J., Liles, K., Chun, M., Li, P., Gohara, D.W., Dolinsky, T., Konecny, R., Koes, D.R., Nielsen, J.E., Head-Gordon, T., Geng, W., Krasny, R., Wei, G.-W., Holst, M.J., McCammon, J.A. and Baker, N.A., Improvements to the APBS biomolecular solvation software suite. *Protein Sci* **2018**, 27, 112-128.

(6) Morrison, J. F., Kinetics of the reversible inhibition of enzyme-catalysed reactions by tight-binding inhibitors. *Biochim Biophys Acta* **1969**, *185* (2), 269-86.

(7) Murphy, D. J., Determination of Accurate KI Values for Tight-binding Enzyme Inhibitors: an in Silico Study of Experimental Error and Assay Design. *Anal Biochem* **2004**, *327* (1), 61-7.

(8) Xu X., Clément, P., Eklöf-Österberg, J., Kelley-Loughnane, N., Moth-Poulsen, K., Chávez, Jorge L., Palma, M., Reconfigurable Carbon Nanotube Multiplexed Sensing Devices. *Nano Lett* **2018**, *18* (7), 4130-4135.
